# Endocrowns for Rehabilitation of Anterior Teeth: *In Vitro* Mechanical Analysis

**DOI:** 10.1055/s-0045-1811715

**Published:** 2025-09-22

**Authors:** Alison Flávio Campos dos Santos, Kusai Baroudi, Rama Yassin Almaghribi, Laís Regiane Silva Concílio, Regina Clara Gambaro de Abreu, Marina Amaral

**Affiliations:** 1 Postgraduate Program in Health Sciences, University of Taubaté, Taubaté, Brazil; 2 Department of Clinical Sciences, College of Dentistry, Ajman University, Ajman, United Arab Emirates; 3 Centre of Medical and Bio-allied Health Sciences Research, Ajman, United Arab Emirates; 4 Department of Dentistry, University of Taubaté, Taubaté, Brazil

**Keywords:** crowns, ceramics, lithium disilicate, fatigue

## Abstract

**Objective:**

This article evaluates the fracture load after mechanical cycling of severely damaged endodontically treated teeth restored with: (1) fiber post, composite resin core, and lithium disilicate (LD) crown; (2) individually fabricated LD post-core and LD crown; (3) LD endocrown; or (4) resin matrix ceramic endocrown.

**Materials and Methods:**

Sixty bovine roots were endodontically treated and prepared for intraradicular retention at depths of 10 or 5 mm. Fiber posts or individually fabricated LD post-cores were cemented into 10-mm-deep prepared root canals. LD crowns were manufactured and cemented onto the cores. Endocrowns (LD or resin matrix ceramic) were fabricated and cemented into 5-mm-deep prepared roots. All samples ( *n* = 15) were subjected to mechanical cycling (1 × 10 ^6^ cycles at 100 N and 4 Hz), followed by fracture load testing and failure mode analysis.

**Statistical Analysis:**

Data were subjected to Kruskal–Wallis followed by Dwass-Steel-Critchlow-Fligner test ( *α* = 0.05).

**Results:**

The LD post-core group exhibited seven failures regarding endodontic retention during mechanical cycling and showed the lowest fracture load (192.9 N; *p* = 0.021). The highest fracture load was observed in the resin matrix ceramic endocrown group (713.9 N), with three catastrophic failures (root fracture). The fiber post-resin core-crown group presented the lowest number of failures during fatigue test (13%) and the lowest number of catastrophic failures (13% root fracture).

**Discussion:**

The improved bonding potential of resin matrix ceramic endocrowns may contribute to higher fracture resistance and enhanced survival under mechanical fatigue compared to LD post-core systems.

**Conclusion:**

Resin matrix ceramic endocrowns are an option for restoring anterior severely damaged endodontically treated teeth, with the highest load to failure. However, the conventional post-core-crown strategy demonstrated lower number of failures during fatigue and lowest number of catastrophic failures (root fracture).

## Introduction

The absence of a ferrule—defined as the presence of 1.5 to 2 mm of coronal dentin after tooth preparation—represents a significant challenge in the rehabilitation of severely damaged teeth. 1 These teeth have typically undergone root canal treatment, and their restoration often requires intraradicular retention to support a core buildup. 2 Posts are generally indicated when less than 50% of the coronal tooth structure remains. 3 However, post-restored anterior teeth exhibit higher fracture rates 4 compared to posterior teeth, likely due to the increased shear forces generated during excursive jaw movements. Consequently, there is a growing trend toward minimizing the use of intraradicular posts in the restoration of endodontically treated teeth.

A conservative and biomechanically favorable option for post-retained restorations involves the use of glass fiber-reinforced composite posts. The elastic modulus of fiber posts is similar to that of dentin, and, when adhesively cemented, they promote more uniform stress distribution, thereby reducing the risk of root fracture. Fiber posts have been shown to improve the fracture resistance of restored teeth. 5 However, recent systematic reviews report similar clinical success rates between metallic and fiber posts, 6 and some studies suggest that fiber posts may even increase the incidence of catastrophic failures. 7 Despite these findings, the post-core-crown approach remains the most widely accepted method for restoring anterior teeth. 8

One of the main concerns in the use of glass fiber posts for the rehabilitation of severely compromised teeth is the risk of post debonding. 9 Adhesion to the root canal walls is particularly challenging due to the high configuration factor (“C-factor”), reduced light transmission in apical regions, and the presence of thick cement layers resulting from the prefabricated nature of fiber posts, which often do not closely adapt to the canal anatomy. A custom-fabricated post-and-core system, using a single material tailored to the individual canal morphology, could minimize the number of interfaces, reduce clinical steps and chair time, 10 and decrease cement thickness—ultimately improving retention. Although metallic post-and-core systems have been used for many years, they are associated with poor esthetics and a higher risk of root fracture. An alternative would be an individualized lithium disilicate (LD) post-and-core. LD ceramic offers both esthetics and strength, sufficient for the indication of three-unit anterior fixed dental prostheses.

On the other hand, advancements in restorative materials and techniques have prompted reconsideration of the necessity of intraradicular posts for the restoration of severely damaged endodontically treated teeth. Moreover, canal preparation for post placement may involve up to two-thirds of the root length, potentially resulting in the loss of up to 58.3% of tooth structure in incisors and increasing the risk of root perforation. 11 12 Minimally invasive endodontic restorative strategies require further investigation to determine the effectiveness of these protocols. The combination of tooth structure preservation, enhanced adhesion, and digital dentistry should be considered in the development of novel restorative techniques. 13

Given these concerns, endocrowns have emerged as a promising alternative for the restoration of extensively damaged endodontically treated teeth. Endocrowns are adhesive restorations anchored in the pulp chamber, without the need for root canal preparation. 14 They are well established for posterior teeth, 15 particularly in situations with limited interocclusal space. 16 Compared to post-core-crown restorations, tooth preparation for endocrowns is more conservative and does not involve intervention in the treated root canals. 17 Endocrowns have demonstrated similar or superior biomechanical performance compared to traditional post-core-crown restorations, including in anterior teeth. 18 These restorations must be fabricated from adhesive-compatible materials, such as glass matrix ceramics (e.g., feldspathic, leucite-reinforced, LD ceramics) or resin composites.

Endocrowns have demonstrated superior mechanical performance and fracture resistance compared to fiber post systems. 8 19 20 Clinical success rates of 87.1% have been reported for posterior endocrowns after 5 years of function, 21 although higher failure rates have been observed in premolars compared to molars after 12 years. 22 When compared with fiber post systems, which show survival rates of 66.7% over 2 years, endocrowns have achieved 100% survival within the same period. 23 However, the anatomical characteristics of anterior teeth—greater height, reduced width, and smaller bonding surface—combined with shear forces during function, 24 present a less favorable scenario for endocrown application in this region.

In an effort to mitigate the effects of shear forces acting on endocrowns in anterior teeth, the selection of the restorative material may represent a relevant clinical strategy. LD ceramic appears to be a suitable choice due to its favorable combination of mechanical strength and esthetics. 14 15 However, its elastic modulus is significantly higher than that of dental tissues, particularly dentin. Therefore, the use of resin matrix ceramics—materials that offer acceptable esthetic properties and adhesive potential, combined with an elastic modulus more closely aligned with dentin—may enhance stress dissipation and promote a more favorable biomechanical environment. 25

Considering the known disadvantages of glass fiber posts—such as loss of retention, risk of root perforation, and the need for extensive canal preparation—this study aimed to evaluate alternative restorative strategies for severely damaged anterior teeth and to compare their performance with the conventional glass fiber post and resin core technique. The null hypothesis was that there would be no significant difference in fracture load after mechanical aging among teeth restored with: (1) fiber post, resin core, and LD crown; (2) LD post-core and LD crown; (3) LD endocrown; or (4) resin matrix ceramic endocrown (RMCEc).

## Material and Methods

Table 1 summarizes the test groups and experimental design of this study.

**Table 1:** Study design and experimental groups

Samples preparation					Ageing	Test	Analysis
Unirradicular bovine teeth ( *N* = 60)	Endodontic treatment	Root canal preparation in 10 mm	Glass fiber post and resin composite core reconstruction	LD conventional crown ( *n* = 15) (FPC)	Mechanical cycling (100 N, 4 Hz)	Load to failure test	One-way analysis of variance Failure analysis
			Individualized LD post/core	LD conventional crown ( *n* = 15) (LDPCC)			
		Root canal preparation in 5 mm	No additional intervention	LD endocrown ( *n* = 15) (LDEc)			
				Resin-matrix ceramic endocrown ( *n* = 15) (RMCEc)			

Abbreviations: FPC, fiber post + crown; LD, lithium disilicate; LDEc, lithium disilicate endocrown; LDPCC, lithium disilicate post/core + crown; RMCEc, resin matrix ceramic endocrown.

### Root and Root Canal Preparation

Single-rooted bovine teeth ( *N* = 60) were obtained from a certified slaughterhouse. The coronal portion of the teeth was sectioned with a precision cutting machine (IsoMet 1000, Buehler, Lake Bluff, Illinois, United States), standardizing the roots at 14 mm, with a flat coronal surface, considering complete absence of a ferrule. Roots were selected according to root canal diameter: root canals larger than the drill referring to the glass fiber post #2 (White post DC #2, FGM, Joinville, Brazil) were replaced by adequate ones. The root canals were instrumented with rotary files (U-File, TDK aFILES, Gravataí, Brazil), with a smooth in-and-out movement using a specific motor (Silver Reciproc, VDW, Charlotte, North Carolina, United States) according to the manufacturer's instructions. The instrumentation sequence was T file glide path at 300 revolutions per minute (RPM) 3 N/cm, followed by #25.08 at 300 RPM 3 N/cm until the file reached working length. A final rinse of the root canal was carried out, with Easy Clean agitation of 5 mL of aqueous ethylenediaminetetraacetic acid for 1 minute and 5 mL of 5.25% sodium hypochlorite for 1 minute. The final rinse was performed with 5 mL of distilled water. The root canals were then dried with absorbent paper points. Gutta-percha cones corresponding to the system were used, together with endodontic cement (AH plus, Dentsply, lot 2404000562, Konstanz, Germany) for root filling, and the elements were stored in a humid environment at 37°C.

After 7 days of endodontic treatment, the root canals were prepared for restoration with: (1) fiberglass post associated with a composite resin core and full LD crown, (2) LD post + individualized core, in a single-piece format and full LD crown, (3) LD endocrown, or (4) RMCEc ( *n* = 15).

Root canals of 30 teeth were prepared in 10 mm for the cementation of fiberglass posts and LD post/core, and the other 30 teeth root canals were prepared in 5 mm for the manufacture of endocrowns. Gutta-percha/endodontic cement were removed from root canals with Gates and Lago drills in sequences #1, #2, #3, and #4, followed by the drill referring to the glass fiber post #2 (White post DC #2, FGM). Prepared root canals were irrigated with 1% sodium hypochlorite (Cloro Rio, RIOQUIMICA Industria Farmacêutica, São José do Rio Preto, Brazil) for cleaning.

### Root Retention Cementation Protocol

- Fiber post + crown (FPC): Glass fiber post and resin composite core reconstruction associated to LD crown: 15 ( *n* = 15) roots with root canal prepared in 10 mm, received cementation of a glass fiber post and had the core reconstructed with direct resin composite. The glass fiber posts (White post DC #2, FGM, lot 201119) were treated with 37% phosphoric acid (Condac, FGM, lot 230822) for 1 minute to clean the surface, followed by the application of silane agent (Prosil, FGM, lot 130523). A dual cure self-adhesive resin cement (RelyX U200, 3M ESPE, lot 11196007, Neuss, Germany) was manipulated according to the manufacturer's instructions, and inserted into the canal with a specific syringe (Centrix, Maquira, Maringá, Brazil). The post was immediately positioned into the root canal, the excess resin cement was removed, and the set was light-activated (Bluephase N, Ivoclar Vivadent, Schaan, Liechtenstein) for 60 seconds on the occlusal surface. The post was sectioned at 6 mm from the root canal entrance, in the coronary direction. The coronal portion of the dental element was etched with 37% phosphoric acid (Condac, FGM, lot 230822) for 15 seconds, followed by washing and drying and application of dentin adhesive (Ambar APS, FGM, lot 050521), followed by photoactivatinon (40 seconds, Bluephase N, Ivoclar Vivadent). Coronary core portion were reconstructed with resin composite (Opalis, FGM, lot 090524) using a standardized acetate matrix (with 7 mm in height, 3 mm mesiodistal, and 3 mmm buccolingual dimensions, fabricated from a 0.5-mm thermoformed acetate plate over the full crown preparation of a maxillary central incisor from a simulation mannequin).
- LD post/core + crown (LDPCC): 15 roots with root canal prepared in 10 mm, had the root canal modeled with the aid of standard pins (Pinjet, Agelus, Londrina, Brazil) and low contraction acrylic resin (Duralay Reliance, Dental MFG, Santo Amaro, Brazil). The coronal excess of the standard post was sectioned at a height of 6 mm from the root canal entrance, and the core portion was reconstructed also in low contraction acrylic resin with standardized acetate matrices, as described before. The acrylic resin patterns were sent to the prosthetic laboratory to the fabrication of a post/core piece in pressed LD (e.max PRESS, Ivoclar Vivadent). For cementation, the LD posts were treated with 10% hydrofluoric acid (Condac Porcelain, FGM, lot 230822) in the post position, for 20 seconds, followed by abundant rinse and drying, and application of silane agent (Prosil, FGM, lot 130523). A dual cure self-adhesive resin cement (RelyX U200, 3M ESPE, lot 11196007) was manipulated according to the manufacturer's instructions, and inserted into the canal with a specific syringe (Centrix, Maquira). The post/core was immediately positioned inside the root canal, excess resin cement was removed, and the set was light-activated (Bluephase N, Ivoclar Vivadent) for 60 seconds on the occlusal surface.
- LD endocrown (LDEc) and RMCEc: 30 roots had their root canal prepared in 5 mm, so that the cavity would promote retention for the endocrown. Preparation was performed with the drill referring to the glass fiber post #2 (White post DC #2, FGM), resulting in a circular cavity, with 1.2 mm diameter and 5 mm apical depth.

### Milling of Restorations

**Fig. 1:**
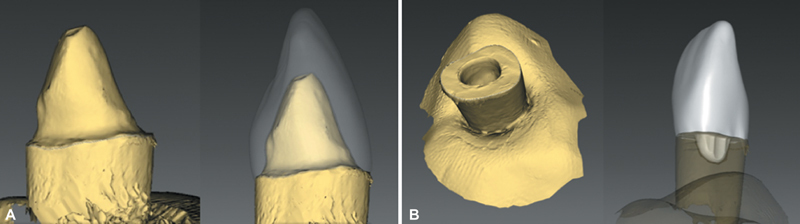
( **A** ) Scanning of sample with post-core assemblies (left) and planned full crown (right). ( **B** ) Scanning of sample prepared for endocrown (left) and planned endocrown (right).

All prepared elements were taken to a bench scanner (inEos Blue, Sirona, Bensheim, Germany), for scanning the preparations and transfer the images to a computer-aided design (CAD) software (InLab, Sirona) ( Fig. 1A ). For teeth with fiberglass posts (FPC group) or LD posts/cores (LDPCC group), conventional full crowns were made, with 1 mm axial thickness and 2 mm incisal thickness, with the 30 crowns being milled (CEREC, Sirona) in LD (e.max CAD, Ivoclar Vivadent). Endocrowns were fabricated for LDEc and RMCEc, with approximate cervico-incisal and mesiodistal dimensions as the previous crowns. Half of endocrowns ( *n* = 15) were milled in LD (LDEc group), and the other half ( *n* = 15) were milled in resin matrix ceramic (RMCEc group) (Vita Enamic, Vita ZahnFabrik, lot 39560, Bad Säckingen, Germany) ( Fig. 1B ). All crowns were finished as specified by the manufacturer, and the LD crowns were crystallized in a specific oven.

### Crown and Endocrowns Cementation

The crowns were cemented onto the dental elements using an adhesive protocol:

- FPC: The internal portion of LD crowns were etched with 10% hydrofluoric acid (Condac porcelain, FGM, lot 230822) for 20 seconds, and silane was applied (Prosil, FGM, lot 130523) for 60 seconds. The universal adhesive system (Adper SingleBond Universal, 3M ESPE, lot 2423400204, Bad Sackingen, Germany) was applied to dentin and resin composite core and cervical dentin. A conventional dual cure resin cement (RelyX Ultimate, 3M ESPE) was manipulated, inserted inside the crowns and the restorations were immediately positioned over the prepared roots. A manual load was applied to the crowns for full seating (until the restoration margins joined with the end of tooth preparation) and drainage of excess cement. Excess cement was removed and photoactivation was performed (Bluephase N, Ivoclar Vivadent) for 30 seconds on the buccal and lingual surfaces of the elements.
- LDPCC: The internal portion of LD crowns were etched with 10% hydrofluoric acid (Condac porcelain, FGM, lot 230822) for 20 seconds, and silane was applied (Prosil, FGM, lot 130523) for 60 seconds. The LD core cemented to root canal was conditioned with 10% hydrofluoric acid (Condac Porcelain, FGM, lot 230822) for 20 seconds, followed by abundant rinse and drying. The universal adhesive system (Adper SingleBond Universal, 3M ESPE, lot 2423400204) was applied to LD core and root cervical dentin. The internal portion of LD crowns were etched with 10% hydrofluoric acid (Condac porcelain, FGM, lot 230822) for 20 seconds, and silane was applied (Prosil, FGM, lot 130523) for 60 seconds. A conventional dual cure resin cement (RelyX Ultimate, 3M ESPE) was manipulated, inserted inside the crowns, and the restorations were immediately positioned over the prepared roots. A manual load was applied to the crowns for full seating (until the restoration margins joined with the end of tooth preparation) and drainage of excess cement. Excess cement was removed and photoactivation was performed (Bluephase N, Ivoclar Vivadent) for 30 seconds on the buccal and lingual surfaces of the elements.
- LDEc: LDEc received conditioning with 10% hydrofluoric acid (Condac Porcelain, FGM, lot 230822) in the intraradicular retention and cervical portion for 20 seconds, and application of silane (Prosil, FGM, lot 130523) for 60 seconds. The universal adhesive system (Adper SingleBond Universal, 3M ESPE, lot 2423400204) was applied to root dentin. A conventional dual cure resin cement (RelyX Ultimate, 3M ESPE) was manipulated, inserted inside the crowns, and the restorations were immediately positioned over the prepared roots. A manual load was applied to the crowns for full seating (until the restoration margins joined with the end of tooth preparation) and drainage of excess cement. Excess cement was removed and photoactivation was performed (Bluephase N, Ivoclar Vivadent) for 30 seconds on the buccal and lingual surfaces of the elements.
- RMCEc: RMCEc received conditioning with 10% hydrofluoric acid (Condac Porcelain, FGM, lot 230822) in the intraradicular retention and cervical portion for 60 seconds, and application of silane (Prosil, FGM, lot 130523) for 60 seconds. The universal adhesive system (Adper SingleBond Universal, 3M ESPE, lot 2423400204) was applied to root dentin. A conventional dual cure resin cement (RelyX Ultimate, 3M ESPE) was manipulated, inserted inside the crowns, and the restorations were immediately positioned over the prepared roots. A manual load was applied to the crowns for full seating (until the restoration margins joined with the end of tooth preparation) and drainage of excess cement. Excess cement was removed and photoactivation was performed (Bluephase N, Ivoclar Vivadent) for 30 seconds on the buccal and lingual surfaces of the elements.

### Inclusion of Samples and Fatigue Cycling

Afterwards, the roots were included up to 2 mm below the cementation line, in polyvinyl chloride tube matrices (25 mm × 15 mm, Tigre) and filled with chemically activated acrylic resin (Dencôr, Clássico, lot 31503, Campo Limpo Paulista, Brazil). The samples were stored for 7 days in a humid environment at 37°C, and then subjected to mechanical cycling. The samples were positioned in the mechanical cycling machine (BioCycle, BioPDI, Sao Carlos, Brazil) at 45°, immersed into water, and received the application of 1 million load cycles of 100 N, at 4 Hz frequency, on the palatal portion of the teeth. Samples were evaluated once a day for root fracture, crown fracture, or loss of retention of any piece during mechanical cycling.

### Load to Fracture Test and Fracture Analysis

The surviving samples were subjected to the fracture load test on a universal testing machine (Mbio 5000, BioPDI). Samples were positioned at 45° and received an increasing load (1 mm/s) at the palatal surface, until fracture noise or signs (cracks or loss of retention). Fracture analysis was performed, classifying samples with catastrophic (root fracture) or reparable (crown/intraradicular retention fracture or loss of retention) failure. Representative samples were subjected to stereomicroscopic analysis for fracture origin identification (Discovery V20, Karl Zeiss).

### Sample Size and Statistical Analysis

Sample size was stipulated based on previous researches, 26 aiming to perform Weibull analysis after test. Due to the number of lost samples during fatigue, the Shapiro–Wilk test indicated a nonparametrical distribution of data ( *p* -values: FPC = 0.489; LDPCC = 0.934; LDEc = 0.007; RMCEc = 0.402). Kruskal–Wallis followed by Dwass-Steel-Critchlow-Fligner test for groups comparison of load-to-failure test at the significance level of 95%.

## Results

Table 2 shows the number and classification of failures (dropouts) during mechanical cycling. The FPC group presented the lowest number of failures during mechanical cycling (2 failures due to crown loss of retention), whereas the LDPCC group exhibited the highest number of failures (7 failures due to fracture of the intraradicular retention). No root fractures were recorded during mechanical cycling.

**Table 2:** Number and classification of failures during mechanical cycling

	Crown loss of retention	Fracture of intraradicular retention	Total of specimens failed
FPC	2		2
LDPCC		7	7
LDEc	2	4	6
RMCEc	6		6

Abbreviations: FPC, fiber post + crown; LDEc, lithium disilicate endocrown; LDPCC, lithium disilicate post/core + crown; RMCEc, resin matrix ceramic endocrown.

Table 3 presents the load-to-failure data and failure mode analysis after testing. A statistically significant difference was found between groups (chi-square = 33.3; degree of freedom = 3; *p* < 0.001), rejecting the null hypothesis. The LDPCC group showed the lowest load-to-failure values (median = 185.1 N), with all failures occurring at the intraradicular retention. The RMCEc group showed the highest load-to-failure values (median = 713.9 N), with most failures related to loss of retention both in fatigue and load-to-failure test (repairable failures).

**Table 3:** Failure load data, statistical significance, and failure analysis

	Number of tested samples	Mean load to failure (N)	Median (N) (SE)	Failure analysis		
				Reparable		Catastrophic
				Loss of retention (post/crown)	Crown/ intraradicular retention fracture	Root fracture
FPC	13	677.0	700.9 (114)B	7	4	2
LDPCC	8	192.9	185.1 (44.9)D	0	8	0
LDEc	8	608.0	467.8 (138)C	0	5	3
RMCEc	9	615.0	713.9 (125)A	4	2	3

Abbreviations: FPC, fiber post + crown; LDEc, lithium disilicate endocrown; LDPCC, lithium disilicate post/core + crown; RMCEc, resin matrix ceramic endocrown; SE, standard error.

Note: Different letters indicate statistical difference.

**Fig. 2:**
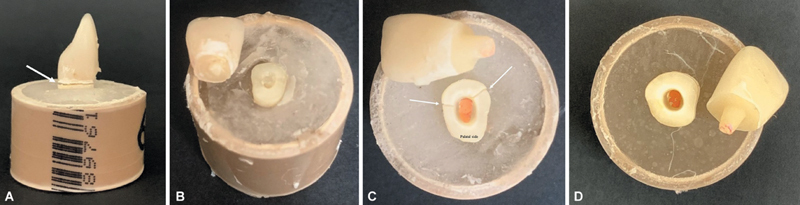
Representative images of each failure mode. ( **A** ) Crown loss of retention in the fiber post + crown (FPC) group (white arrow indicates fracture line at cervical portion). ( **B** ) Fracture of the intraradicular retention in group lithium disilicate post/core + crown (LDPCC). ( **C** ) Root fracture (white arrows at buccal face) associated to crown loss of retention in group lithium disilicate endocrown (LDEc). ( **D** ) Crown loss of retention in group resin matrix ceramic endocrown (RMCEc).

**Fig. 3:**
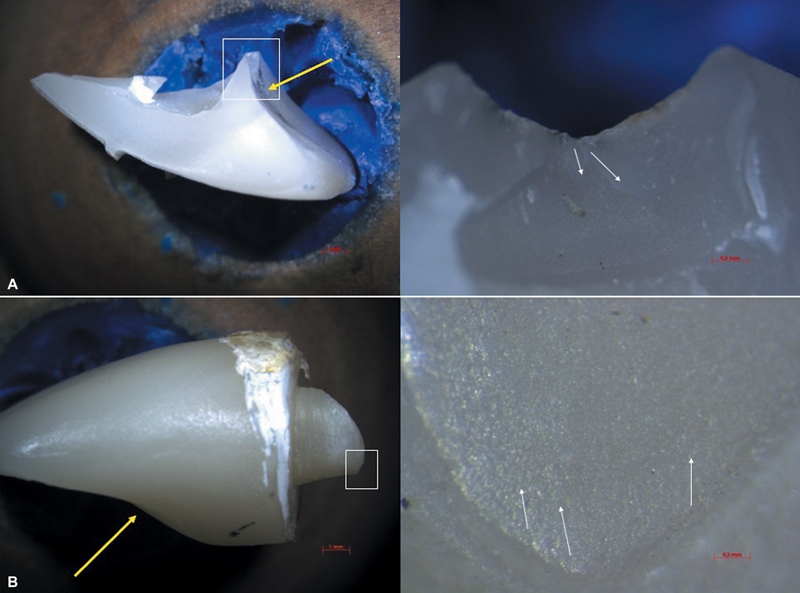
Stereomicroscopic images from failure origin. ( **A** ) The fiber post + crown (FPC) group with failure origin at load application site, at palatal surface of lithium disilicate (LD) crown (7.5× magnification left; 30× magnification right—yellow arrow indicates the load application site. White square indicates the amplification area. White arrows indicate the propagation direction of fracture.) ( **B** ) The lithium disilicate endocrown (LDEc) group with failure origin at the palatal side of the intraradicular extension of the endocrown (7.5× magnification left; 100× magnification right—yellow arrow indicates the load application site. White square indicates the amplification area. White arrows indicate the propagation direction of fracture).

Catastrophic failures (root fractures) occurred only during the load-to-failure test, predominantly in the endocrown groups (20% of failures in both LDEc and RMCEc groups). Fig. 2 presents one representative sample from each group, illustrating the observed failure patterns. Fig. 3 represents microscopic images from fracture origin sites of LD crown ( Fig. 3A , FPC group), at load application site—palatal side, and LDEc ( Fig. 3B , LDEc group) at intraradicular extension, at the palatal side.

## Discussion

The null hypothesis that no significant difference would be observed in fracture load among the tested groups was rejected, as statistically significant differences were identified. RMCEc in anterior teeth exhibited the highest load-to-failure values ( Table 3 ), associated with 3 root fractures and 10 cases of crown loss of retention—6 of which occurred during mechanical cycling. The glass fiber post group combined with a resin composite core and LD crown (FPC) demonstrated the second highest load-to-failure values ( Table 3 ), with only two repairable failures during fatigue testing and two root fractures following the load-to-failure test. The complete absence of a ferrule, as simulated in the present study, represents one of the most challenging clinical conditions for rehabilitation. In such scenarios, alternative approaches—such as cast post-and-core systems or even implant-supported restorations—may be considered.

The FPC group may be considered as presenting the best mechanical performance, based on load-to-failure values, the number of failures during mechanical fatigue, and the failure mode ( Figs. 2A and 3A ). Most failures were repairable, which enhances the clinical survival potential of this restorative strategy. In the present study, the majority of failures in the FPC group (86.6%, Fig. 2A ) were classified as repairable. Fiber posts exhibit clinical performance comparable to that of metal posts. 6 After 9 years, the clinical survival rates are similar between fiber and metal posts; however, fiber posts tend to show a lower success rate, meaning they require more interventions—usually involving recementation or replacement of the post and crown. 9 Root fractures reported in the literature for the fiber post-core-crown approach are typically associated with excessive and horizontally directed occlusal forces, which generate tensile and shear stresses, especially in the absence of remaining coronal walls, 4 as simulated in the present study.

The second strategy using intraradicular retention, tested in the LDPCC group, did not present a feasible strategy, since 46.6% of the samples presented fracture of LD post (intraradicular retention) during fatigue test ( Table 2 ). This group presented no catastrophic failure, probably due to the lowest load-to-failure values presented ( Table 3 )—catastrophic failures are usually associated to high loads applied. 25 Average bite forces—ranging between 20 to 1000 N, not exceeding 270 N during normal function 27 —are similar to fatigue load used (100 N) and load-to-failure presented by the LDPCC group (185.1 N) is below the limit reported for normal function. As a reference, the minimum thickness of 2 mm is necessary for restoration longevity for ceramic onlay. 28 Since root canal was prepared with the fiber post drill, the post space diameter was between 1.05 mm (apical) and 1.8 mm (cervical), the thickness of the tested intraradicular retention could be wider, but it may compromise dentin thickness.

Endocrowns concentrate less tensile stresses in the tooth compared to full crown, with most stress concentrating in the resin endocrown. 29 Another point to be considered regarding fiber post/resin core and endocrown would be the reversibility of treatment, in case of endodontic reintervention necessity. The removal of fiber posts from root canal decreases the fracture resistance value of restored tooth compared to teeth where fiber post was not removed. 30 In this scenario, endocrowns would present an additional advantage.

In the present study, the endocrowns in resin matrix ceramic (RMCEc) presented performance superior to the FPC group considering the load-to-failure values ( Table 3 ). Loss of retention (reparable) was the most frequent failure mode ( Table 3 ) in both fatigue and load-to-failure testes. The superior mechanical performance of RMCEc may reflect a more favorable stress distribution, attributable to their elastic modulus and adhesive integration with dental tissues. 31 The increase in bond strength of RMCEc could result in higher load to failure and survival after mechanical cycling than the presented. Thus, sandblasting the material surface may be recommended for resin matrix ceramics since it removes smear layer, creates a clean surface for retention, and increases surface area and roughening (micromechanical retention). 32

Compared to LDEc, RMCEc also presented highest load-to-failure ( Table 3 ). Less loss of retention was reported for LDEc, but 60% of failures were related to fracture of the intraradicular portion of the endocrown ( Tables 2 and 3 ; Fig. 3B ). Such failure mode may be associated with the mismatch between the elastic modulus of LD and dentin, leading to increased stress concentration and early failure. Besides that, brittle nature of ceramics and low fracture toughness of glass ceramic also contribute to fracture of LDEc. 33

The use of different cements in the present experiment may be explained by: (1) self-adhesive resin cement was used for the cementation of both fiber posts and LD post-and-core restorations, as the use of conventional resin cement combined with a separate adhesive system is less favorable in the root canal environment. This is primarily due to the difficulties mentioned before and during cementation of fiber posts. Additionally, self-adhesive resin cements provide mechanical retention through hygroscopic expansion during polymerization, which contributes to improved adaptation within the canal. And (2), for crown cementation, the combination of Single Bond Universal adhesive and RelyX Ultimate resin cement was selected due to the variability of substrates across groups—such as dentin, composite resin core, and LD ceramic. Single Bond Universal is a self-etching adhesive that contains silane, enabling effective bonding to both dental tissues and ceramic materials. Besides that, it does not require separate activation, since it is chemically activated by the dual-cure resin cement.

The results of this study must be interpreted with caution, since it is an *in vitro* study, and there are few studies evaluating endocrowns for anterior teeth, which limits comparisons and embracing discussion of the issue. The use of bovine teeth is well documented in similar studies, but may overestimate load results since bovine roots tend to present thicker dentin walls. Authors also opted for not simulating periodontal ligament—it simulates more closely clinical situation but turns mechanical cycling hard to perform. Future studies should focus in determining a more biomechanically favorable format for preparation of root canal for a LD post/core.

Although endocrowns are primarily indicated for posterior teeth, the results of this study suggest their potential viability in anterior teeth, particularly when using resin matrix ceramics. However, anatomical limitations—such as reduced bonding area and higher shear forces—must be considered. Notably, catastrophic failures occurred only in endocrown groups and exclusively during load-to-failure testing, highlighting the importance of careful material selection and clinical planning.

## Conclusion

RMCEc present good mechanical performance with minimal endodontic intervention to restore severely destroyed anterior endodontically treated teeth,
